# Automated Detection and Diagnosis of Diabetic Retinopathy: A Comprehensive Survey

**DOI:** 10.3390/jimaging7090165

**Published:** 2021-08-27

**Authors:** Vasudevan Lakshminarayanan, Hoda Kheradfallah, Arya Sarkar, Janarthanam Jothi Balaji

**Affiliations:** 1 Theoretical and Experimental Epistemology Lab, School of Optometry and Vision Science, University of Waterloo, Waterloo, ON N2L 3G1, Canada; hkheradfallah@uwaterloo.ca; 2 Department of Computer Engineering, University of Engineering and Management, Kolkata 700 156, India; aryasarkarwork@gmail.com; 3 Department of Optometry, Medical Research Foundation, Chennai 600 006, India; jothibalaji@gmail.com

**Keywords:** diabetic retinopathy, artificial intelligence, deep learning, machine-learning, datasets, fundus image, optical coherence tomography, ophthalmology

## Abstract

Diabetic Retinopathy (DR) is a leading cause of vision loss in the world. In the past few years, artificial intelligence (AI) based approaches have been used to detect and grade DR. Early detection enables appropriate treatment and thus prevents vision loss. For this purpose, both fundus and optical coherence tomography (OCT) images are used to image the retina. Next, Deep-learning (DL)-/machine-learning (ML)-based approaches make it possible to extract features from the images and to detect the presence of DR, grade its severity and segment associated lesions. This review covers the literature dealing with AI approaches to DR such as ML and DL in classification and segmentation that have been published in the open literature within six years (2016–2021). In addition, a comprehensive list of available DR datasets is reported. This list was constructed using both the PICO (P-Patient, I-Intervention, C-Control, O-Outcome) and Preferred Reporting Items for Systematic Review and Meta-analysis (PRISMA) 2009 search strategies. We summarize a total of 114 published articles which conformed to the scope of the review. In addition, a list of 43 major datasets is presented.

## 1. Introduction

Diabetic retinopathy (DR) is a major cause of irreversible visual impairment and blindness worldwide [1]. This etiology of DR is due to chronic high blood glucose levels, which cause retinal capillary damage, and mainly affects the working-age population. DR begins at a mild level with no apparent visual symptoms but it can progress to severe and proliferated levels and progression of the disease can lead to blindness. Thus, early diagnosis and regular screening can decrease the risk of visual loss to 57.0% as well as decreasing the cost of treatment [2].

**Figure 1:**
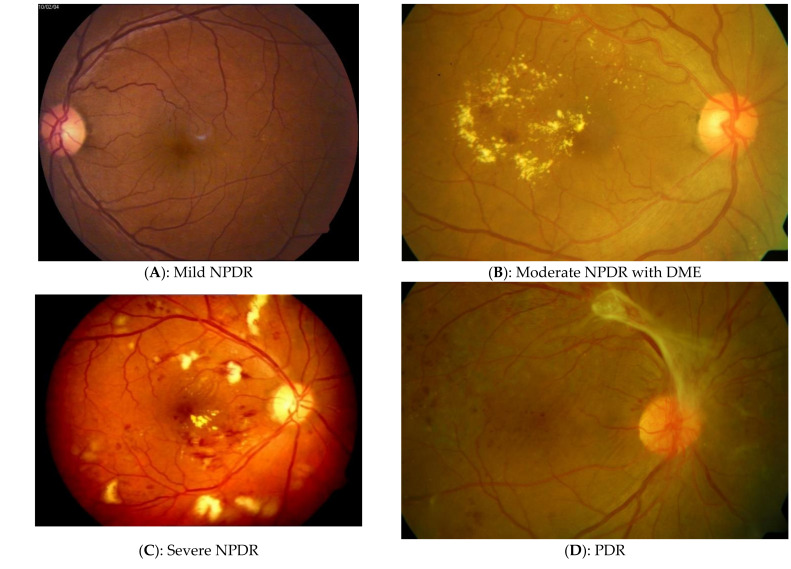
Retinal fundus images of different stages of diabetic retinopathy. (**A**) Stage II: Mild non-proliferative diabetic retinopathy; (**B**) Stage III: Moderate non-proliferative diabetic retinopathy; (**C**) Stage IV: Severe non-proliferative diabetic retinopathy; (**D**) Stage V: Proliferative diabetic retinopathy (images courtesy of Rajiv Raman et al., Sankara Nethralaya, India).

DR is clinically diagnosed through observation of the retinal fundus either directly or through imaging techniques such as fundus photography or optical coherence tomography. There are several standard DR grading systems such as the Early Treatment Diabetic Retinopathy Study (ETDRS) [3]. ETDRS separates fine detailed DR characteristics using multiple levels. This type of grading is done upon all seven retinal fundus Fields of View (FOV). Although ETDRS [4] is the gold standard, due to implementation complexity and technical limitations [5], alternative grading systems are also used such as the International Clinical Diabetic Retinopathy (ICDR) [6] scale which is accepted in both clinical and Computer-Aided Diagnosis (CAD) settings [7]. The ICDR scale defines 5 severity levels and 4 levels for Diabetic Macular Edema (DME) and requires fewer FOVs [6]. The ICDR levels are discussed below and are illustrated in Figure 1.

- No Apparent Retinopathy: No abnormalities.
- Mild Non-Proliferative Diabetic Retinopathy (NPDR): This is the first stage of diabetic retinopathy, specifically characterized by tiny areas of swelling in retinal blood vessels known as Microaneurysms (MA) [8]. There is an absence of profuse bleeding in retinal nerves and if DR is detected at this stage, it can help save the patient’s eyesight with proper medical treatment (Figure 1A).
- Moderate NPDR: When left unchecked, mild NPDR progresses to a moderate stage when there is blood leakage from the blocked retinal vessels. Additionally, at this stage, Hard Exudates (Ex) may exist (Figure 1B). Furthermore, the dilation and constriction of venules in the retina causes Venous Beadings (VB) which are visible ophthalmospically [8].
- Severe NPDR: A larger number of retinal blood vessels are blocked in this stage, causing over 20 Intra-retinal Hemorrhages (IHE; Figure 1C) in all 4 fundus quadrants or there are Intra-Retinal Microvascular Abnormalities (IRMA) which can be seen as bulges of thin vessels. IRMA appears as small and sharp-border red spots in at least one quadran. Furthermore, there can be a definite evidence of VB in over 2 quadrants [8].
- Proliferative Diabetic Retinopathy (PDR): This is an advanced stage of the disease that occurs when the condition is left unchecked for an extended period of time. New blood vessels form in the retina and the condition is termed Neovascularization (NV). These blood vessels are often fragile, with a consequent risk of fluid leakage and proliferation of fibrous tissue [8]. Different functional visual problems occur at PDR, such as blurriness, reduced field of vision, and even complete blindness in some cases (Figure 1D).

DR detection has two main steps: screening and diagnosis. For this purpose, fine pathognomonic DR signs in initial stages are determined normally, after dilating pupils (mydriasis). Then, DR screening is performed through slit lamp bio-microscopy with a + 90.0 D lens, and direct [9]/indirect ophthalmoscopy [10]. The next step is to diagnose DR which is done through finding DR-associated lesions and comparing with the standard grading system criteria. Currently, the diagnosis step is done manually. This procedure is costly, time consuming and requires highly trained clinicians who have considerable experience and diagnostic precision. Even if all these resources are available there is still the possibility of misdiagnosis [11]. This dependency on manual evaluation makes the situation challenging. In addition, in year 2020, the number of adults worldwide with DR, and vision-threatening DR was estimated to be 103.12 million, and 28.54 million. By the year 2045, the numbers are projected to increase to 160.50 million, and 44.82 million [12]. In addition, in developing countries where there is a shortage of ophthalmologists [13,14] as well as access to standard clinical facilities. This problem also exists in underserved areas of the developed world.

Recent developments in CAD techniques, which are defined in the subscope of artificial intelligence (AI), are becoming more prominent in modern ophthalmology [15] as they can save time, cost and human resources for routine DR screening and involve lower diagnostic error factors [15]. CAD can also efficiently manage the increasing number of afflicted DR patients [16] and diagnose DR in early stages when fewer sight threatening effects are present. The scope of AI based approaches are divided between Machine Learning-based (ML) and Deep Learning-based (DL) solutions. These techniques vary depending on the imaging system and disease severity. For instance, in early levels of DR, super-resolution ultrasound imaging of microvessels [17] is used to visualize the deep ocular vasculature. On this imaging system, a successful CAD method applied a DL model for segmenting lesions on ultrasound images [18]. The widely applied imaging methods such as Optical Coherence Tomography (OCT), OCT Angiography (OCTA), Ultrawide-field fundus (UWF) and standard 45° fundus photography are covered in this review. In addition to the mentioned imaging methods, Majumder et al. [15] reported a real time DR screening procedure using a smartphone camera.

The main purpose of this review is to analyze 114 articles published within the last 6 years that focus on the detection of DR using CAD techniques. These techniques have made considerable progress in performance with the use of ML and DL schemes that employ the latest developments in Deep Convolutional Neural Networks (DCNNs) architectures for DR severity grading, progression analysis, abnormality detection and semantic segmentation. An overview of ophthalmic applications of convolutional neural networks is presented in [19,20,21,22].

## 2. Methods

### 2.1. Literature Search Details

For this review, literature from 5 publicly accessible databases were surveyed. The databases were chosen based on their depth, their ease of accessibility, and their popularity. These 5 databases are:

- PubMed: Publications from MEDLINE (https://pubmed.ncbi.nlm.nih.gov/ accessed on date 14 June 2021)
- IEEE Xplore: IEEE conference & journals (https://ieeexplore.ieee.org/Xplore/home.jsp accessed on 14 June 2021)
- PUBLONS: Publications from Web of Science (https://publons.com/about/home/ accessed on 14 June 2021)
- SPIE digital library: Conference & journals from SPIE (https://www.spiedigitallibrary.org/ accessed on 14 June 2021)
- Google Scholar: Conference and journal proceedings from multiple databases (https://scholar.google.co.in/ accessed on 14 June 2021).

**Figure 2:**
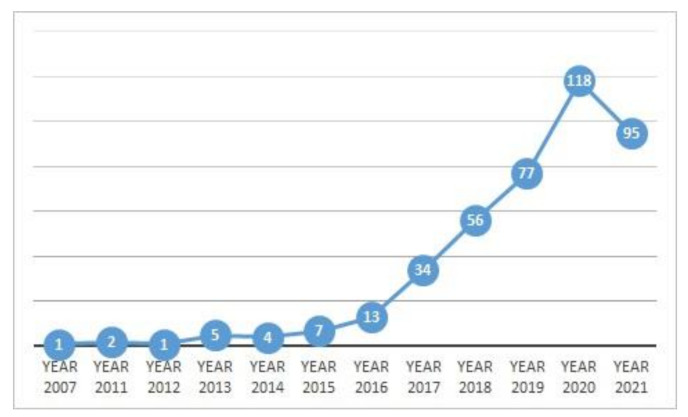
Increase in the number of articles matching the predefined keywords over the last 6 years; the PubMed search results were used to create this figure.

Google Scholar has been chosen to fill the gaps in the search strategy by identifying literature from multiple sources, along with articles that might be missed in manual selection from the other four databases. The articles of this topic within the latest six year time-period show that the advances in AI-enabled DR detection has increased considerably. Figure 2 visualizes the articles matching with this topic. This figure was generated using the PubMed results.

At the time of writing this review, a total of 10,635 search results were listed in the PubMed database for this time period when just the term “diabetic retinopathy” was used. The MEDLINE database is arguably the largest for biomedical research. In addition, some resources from the National Library of Medicine which is a part of the U.S. National Institutes of Health, were employed in this review.

**Figure 3:**
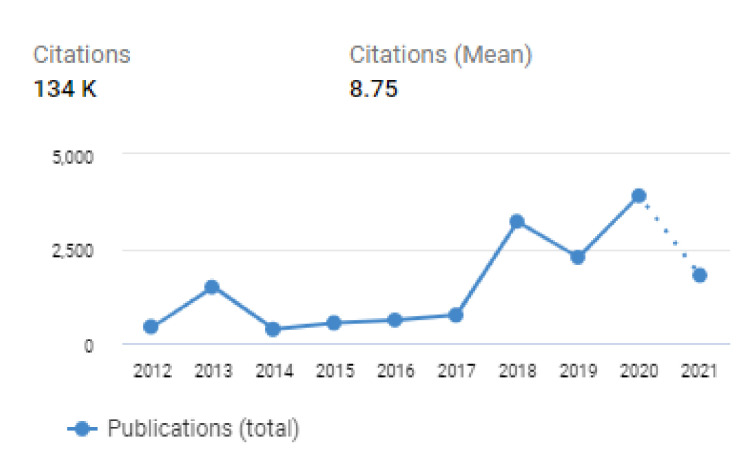
A plot of the number of articles as a function of year. This figure was generated using results from all 5 databases using search terms Diabetic Retinopathy AND (“Deep Learning” OR “Machine Learning”).

A search of the IEEE Xplore library and the SPIE digital library for the given time period reports a total of 812 and 332 search results respectively. The IEEE Xplore and SPIE libraries contain only publications of these two professional societies. Further databases were added to this list by collecting papers from non-traditional sources such as pre-print servers such as ArXiv. In Figure 3, by using data from all sources, we plot the number of papers published as a function of the year.

The scope of this review is limited to “automated detection and grading of diabetic retinopathy using fundus & OCT images”. Therefore, to make the search more manageable, a combination of relevant keywords was applied using the PICO (P-Patient, I-Intervention, C-Control, O-Outcome) search strategy [23]. Keywords used in the PICO search were predetermined. A combination of (“DR” and (“DL” or “ML” or “AI”)) and (fundus or OCT) was used which reduced the initial 10,635 search results in PubMed to just 217 during the period under consideration. A manual process of eliminating duplicate search results carried out across the results from all obtained databases resulted in a total number of 114 papers.

Overall, the search strategy for identifying relevant research for the review involved three main steps:

1. Using the predefined set of keywords and logical operators, a small set of papers were identified in this time range (2016–2021).
2. Using a manual search strategy, the papers falling outside the scope of this review were eliminated.
3. The duplicate articles (i.e., the papers occurring in multiple databases) were eliminated to obtain the set of unique articles.

**Figure 4:**
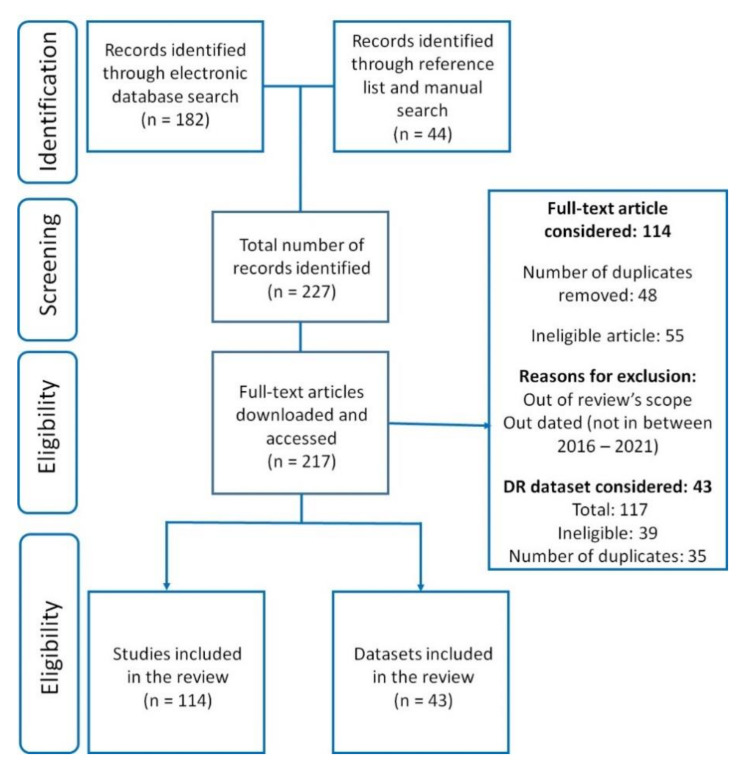
Flowchart summarizing the literature search and dataset identification using PRISMA review strategy for identifying articles related to automated detection and diagnosis of diabetic retinopathy.

The search strategy followed by this review abides by the Preferred Reporting Items for Systematic Review and Meta-analysis (PRISMA) 2009 checklist [24], and the detailed search and identification pipeline is shown in Figure 4.

### 2.2. Dataset Search Details

The backbone of any automated detection model whether ML-based, DL-based, or multi-model-based, is the dataset. High-quality data with correct annotations have extreme importance in image feature extraction and training the DR detection model, properly. In this review, a comprehensive list of datasets has been created and discussed. A previously published paper [25] also gives a list of ophthalmic image datasets, containing 33 datasets that can be used for training DR detection and grading models. The paper by Khan et al. [25] highlighted 33 of the 43 datasets presented in Table 1. However, some databases which are popular and publicly accessible are not listed by Khan et al. [25], e.g., UoA-DR [26], Longitudinal DR screening data [27], FGADR [28] etc. In this review, we identified additional datasets that are available to use. The search strategy for determining relevant DR detection datasets is as follows:

Appropriate results from all 5 of the selected databases (PubMed, PUBLONS, etc.) were searched manually. We gathered information about names of datasets for DR detection and grading.

4. The original papers and websites associated with each dataset were analyzed and a systematic, tabular representation of all available information was created.
5. The Google dataset search and different forums were checked for missing dataset entries and step 2 was repeated for all original datasets found.
6. A final comprehensive list of datasets and its details was generated and represented in Table 1.

A total of 43 datasets were identified employing the search strategy given above. Upon further inspection, a total number of 30 datasets were identified as open access (OA), i.e., can be accessed easily without any permission or payment. Of the total number of datasets, 6 have restricted access. However, the databases can be accessed with the permission of the author or institution; the remaining 7 are private and cannot be accessed. These datasets were used to create a generalized model because of the diversity of images (multi-national and multi-ethnic groups).

## 3. Results

### 3.1. Dataset Search Results

This section provides a high-level overview of the search results that were obtained using the datasets, as well as using different review articles on datasets in the domain of ophthalmology, e.g., Khan et al. [25]. Moreover, different leads obtained from GitHub and other online forums are also employed in this overview. Thus, 43 datasets were identified and a general overview of the datasets is systematically presented in this section. The datasets reviewed in this article are not limited to 2016 to 2021 and could have been released before that. The list of datasets and their characteristics are shown in Table 1 below. Depending on the restrictions and other proforma required for accessing the datasets, the list has been divided into 3 classes; they are:

- Public open access (OA) datasets with high quality DR grades.
- DR datasets, that can be accessed upon request, i.e., can be accessed by filling necessary agreements and forms for fair usage; they are a sub-type of (OA) databases and are termed Access Upon Request (AUR) in the table.
- Private datasets from different institutions that are not publicly accessible or require explicit permission can access are termed Not Open Access (NOA).

### 3.2. Diabetic Retinopathy Classification

This section discusses the classification approaches used for DR detection. The classification can be for the detection of DR [68], referable DR (RDR) [66,69], vision threatening DR (vtDR) [66], or to analyze the proliferation level of DR using the ICDR system. Some studies also considered Diabetic Macular Edema (DME) [69,70]. Recent ML and DL methods have produced promising results in automated DR diagnosis.

Thus, multiple performance metrics such as accuracy (ACC), sensitivity (SE) or recall, specificity (SP) or precision, area under the curve (AUC), F1 and Kappa scores are used to evaluate the classification performance. Table 2 and Table 3 present a brief overview of articles that used fundus images for DR classification, and articles that classify DR on fundus images using novel preprocessing techniques, respectively. Table 4 lists the recent DR classification studies that used OCT and OCTA images. In the following subsections, we provide the details of ML and DL aspects and evaluate the performance of prior studies in terms of quantitative metrics.

#### 3.2.1. Machine Learning Approaches

In this review, 9 out of 93 classification-based studies employed machine learning approaches and 1 article used un-ML method for detecting and grading DR. Hence, in this section, we present an evaluation over various ML-based feature extraction and decision-making techniques that have been employed in the selected primary studies to construct DR detection models. In general, six major distinct ML algorithms were used in these studies. These are: principal component analysis (PCA) [70,71], linear discriminant analysis (LDA)-based feature selection [71], spatial invariant feature transform (SIFT) [71], support vector machine (SVM) [16,71,72,73], k nearest neighbor (KNN) [72] and random forest (RF) [74].

In addition to the widely used ML methods, some studies such as [75] presented a pure ML model with an accuracy of over 80% including distributed Stochastic Neighbor Embedding (t-SNE) for image dimensionality reduction in combination with ML Bagging Ensemble Classifier (ML-BEC). ML-BEC improves classification performance by using the feature bagging technique with a low computational time. Ali et al. [57] focused on five fundamental ML models, named sequential minimal optimization (SMO), logistic (Lg), multi-layer perceptron (MLP), logistic model tree (LMT), and simple logistic (SLg) in the classification level. This study proposed a novel preprocessing method in which the Region of Interest (ROI) of lesions is segmented with the clustering-based method and K-Means; then, Ali et al. [57] extracted features of the histogram, wavelet, grey scale co-occurrence, and run-length matrixes (GLCM and GLRLM) from the segmented ROIs. This method outperformed previous models with an average accuracy of 98.83% with the five ML models. However, an ML model such as SLg performs well; the required classification time is 0.38 with Intel Core i3 1.9 gigahertz (GHz) CPU, 64-bit Windows 10 operating system and 8 gigabytes (GB) memory. This processing time is higher than previous studies.

We can also use ML method for OCT and OCTA for DR detection. Recently, LiU et al. [76] deployed four ML models of logistic regression (LR), logistic regression regularized with the elastic net penalty (LR-EN), support vector machine (SVM), and the gradient boosting tree named XGBoost with over 246 OCTA wavelet features and obtained ACC, SE, and SP of 82%, 71%, and 77%, respectively. This study, despite inadequate results, has the potential to reach higher scores using model optimization and fine-tuning hyper parameters. These studies show a lower overall performance if using a small number of feature types and simple ML models are used. Dimensionality reduction is an application of ML models which can be added in the decision layer of CAD systems [77,78].

The ML methods in combination with DL networks can have a comparable performance with the pure DL models. Narayanan et al. [78] applied a SVM model for the classification of features obtained from the state of art DNNs that are optimized with PCA [78]. This provided an accuracy of 85.7% on preprocessed images. In comparison with methods such as AlexNet, VGG, ResNet, and Inception-v3, the authors report an ACC of 99.5%. In addition, they also found that this technique is more applicable with considerably less computational cost.

#### 3.2.2. Deep Learning Approaches

This section gives an overview of DL algorithms that have been used. Depending on the imaging system, image resolution, noise level, and contrast as well as the size of the dataset, the methods can vary. Some studies propose customized networks such as the work done by Gulshan et al. [69], Gargeya et al. [68], Rajalakshmi et al. [79], Riaz et al. [80]. These networks have lower performance outcomes than the state of art networks such as VGG, ResNet, Inception, and DenseNet but the fewer layers make them more generalized, suitable for training with small datasets, and computationally efficient. Quellec et al. [81] applied L2 regularization over the best performed DCNN in the KAGGLE competition for DR detection named o-O. Another example of customized networks is the model proposed by Sayres et al. [82], which showed 88.4%, 91.5%, 94.8% for ACC, SE, and SP, respectively, over a small subset of 2000 images obtained from the EyePACS database. However, the performance of this network is lower than the results obtained from Mansour et al. [72] which used a larger subset of the EyePACS images (35,126 images). Mansour et al. [72] deployed more complex architectures such as the AlexNet on the extracted features of LDA and PCA that generated better results than Sayres et al. [82] with 97.93%, 100%, and 93% ACC, SE, and SP, respectively. Such DCNNs should be used with large datasets since the large number of images used in the training reduces errors. If a deep architecture is applied for a small number of observations, it might cause overfitting in which the performance over the test data is not as well as expected on the training data. On the other hand, the deepness of networks does not always guarantee higher performance, meaning that they might face problems such as vanishing or exploding gradient which will have to be addressed by redesigning the network to simpler architectures. Furthermore, the deep networks extract several low and high-level features. As these image features get more complicated, it becomes more difficult to interpret. Sometimes, high-level attributes are not clinically meaningful. For instance, the high-level attributes may refer to an existing bias in all images belonging to a certain class, such as light intensity and similar vessel patterns, that are not considered as a sign of DR but the DCNN will consider them as critical features. Consequently, this fact makes the output predictions erroneous.

In the scope of DL-based classification, Hua et al. [83] designed a DL model named Trilogy of Skip-connection Deep Networks (Tri-SDN) over the pretrained base model ResNet50 that applies skip connection blocks to make the tuning faster yielding to ACC and SP of 90.6% and 82.1%, respectively, which is considerably better than the values of 83.3% and 64.1% compared with the situation when skip connection blocks are not used.

There are additional studies that do not focus on proposing new network architectures but enhance the preprocessing step. The study done by Pao et al. [84] presents bi-channel customized CNN in which an image enhancement technique known as unsharp mask is used. The enhanced images and entropy images are used as the inputs of a CNN with 4 convolutional layers with results of 87.83%, 77.81%, 93.88% over ACC, SE, and SP. These results are all higher than the case of analysis without preprocessing (81.80% 68.36%, 89.87%, respectively).

Shankar et al. [85] proposed another approach to preprocessing using Histogram-based segmentation to extract regions containing lesions on fundus images. As the classification step, this article utilized the Synergic DL (SDL) model and the results indicated that the presented SDL model offers better results than popular DCNNs on MESSIDOR 1 database in terms of ACC, SE, SP.

Furthermore, classification is not limited to the DR detection and DCNNs can be applied to detect the presence of DR-related lesions such as the study reported by Wang et al. They cover twelve lesions in their study: MA, IHE, superficial retinal hemorrhages (SRH), Ex, CWS, venous abnormalities (VAN), IRMA, NV at the disc (NVD), NV elsewhere (NVE), pre-retinal FIP, VPHE, and tractional retinal detachment (TRD) with average precision and AUC 0.67 and 0.95, respectively; however, features such as VAN have low individual detection accuracy. This study provides essential steps for DR detection based on the presence of lesions that could be more interpretable than DCNNs which act as black boxes [86,87,88].

There are explainable backpropagation-based methods that produce heatmaps of the lesions associated with DR such as the study done by Keel et al. [89], which highlights Ex, HE, and vascular abnormalities in DR diagnosed images. These methods have limited performance providing generic explanations which might be inadequate as clinically reliable. Table 2, Table 3 and Table 4 briefly summarizes previous studies on DR classification with DL methods.

### 3.3. Diabetic Retinopathy Lesion Segmentation

The state-of-the-art DR classification machines [68,69] identify referable DR identification without directly taking lesion information into account. Therefore, their predictions lack clinical interpretation, despite their high accuracy. This black box nature of DCNNs is the major problem that makes them unsuitable for clinical application [86,152,153] and has made the topic of eXplainable AI (XAI) of major importance [153]. Recently, visualization techniques such as gradient-based XAI have been widely used for evaluating networks. However, these methods with generic heatmaps only highlight the major contributing lesions and hence are not suitable for detection of DR with multiple lesions and severity. Thus, some studies focused on the lesion-based DR detection instead. In general, we found 20 papers that do segmentation of the lesions, such as MA (10 articles), Ex (9 articles) and IHE, VHE, PHE, IRMA, NV, CWS. In the following sections, we discuss the general segmentation approaches. The implementation details of each article are accessible in Table 5 and Table 6 based on its imaging type.

#### 3.3.1. Machine Learning and Un-Machine Learning Approaches

In general, using ML methods with a high processing speed, low computational cost, and interpretable decisions is preferred to DCNNs. However, the automatic detection of subtle lesions such as MA did not reach acceptable values. In this review, we collected 2 pure ML-involved models and 6 un-ML methods. As reported in a study by Ali Shah at el. [154], they detected MA using color, Hessian and curvelet-based feature extraction and achieved a SE of 48.2%. Huang et al. [155] focused on localizing NV through using the Extreme Learning Machine (ELM). This study applied Standard deviation, Gabor, differential invariant, and anisotropic filters for this purpose and with the final classifier applying ELM. This network performed as well as an SVM with lower computational time (6111 s vs. 6877 s) with a PC running the Microsoft Windows 7 operating system with a Pentium Dual-Core E5500 CPU and 4 GB memory. For the segmentation task, the preprocessing step had a fundamental rule which had a direct effect on the outputs. The preprocessing techniques varied depending on the lesion type and the dataset properties. Orlando et al. applied a combination of DCNN extracted features and manually designed features using image illumination correction, CLAHE contrast enhancement, and color equalization. Then, this high dimensionality feature vector was fed into an RF classifier to detect lesions and achieved an AUC score of 0.93, which is comparable with some DCNN models [81,137,141].

Some studies used un-ML methods for detection of exudates such as that of Kaur et al. [156], who proposed a pipeline consisting of a vessel and optic disk removal step and used a dynamic thresholding method for detection of CWS and Ex. Prior to this study, Imani et al. [157] also did the same process with the focus on Ex on a smaller dataset. In their study, they employed additional morphological processing and smooth edge removal to reduce the detection of CWS as Ex. This article reported the SE and SP of 89.1% and 99.9% and had an almost similar performance compared to Kaur’s results with 94.8% and 99.8% for SE and SP, respectively. Further description of the recent studies on lesion segmentation with ML approach can be found in Table 5 and Table 6.

#### 3.3.2. Deep Learning Approaches

Recent works show that DCNNs can produce promising results in automated DR lesion segmentation. DR lesion segmentation is mainly focused on fundus imaging. However, some studies apply a combination of fundus and OCT. Holmberg et al. [158] proposed a retinal layer extraction pipeline to measure retinal thickness with Unet. Furthermore, Yukun Guo et al. [159] applied DCNNs for avascular zone segmentation from OCTA images and received the accuracy of 87.0% for mild to moderate DR and 76.0% for severe DR.

Other studies mainly focus on DCNNS applied to fundus images which give a clear view of existing lesions on the surface of the retina. Other studies such as Lam et al. [160] deployed state of the art DCNNS to detect the existence of DR lesions in image patches using AlexNet, ResNet, GoogleNet, VGG16, and Inception v3 achieving 98.0% accuracy on a subset of 243 fundus images obtained from EyePACS. Wang et al. [28] also applied Inception v3 as the feature map in combination with FCN 32 s as the segmentation part. They reported SE values of 60.7%, 49.5%, 28.3%, 36.3%, 57.3%, 8.7%, 79.8%, and 0.164 over PHE, Ex, VHE, NV, CWS, FIP, IHE, MA, respectively. Quellec et al. [81] focused on four lesions CWS, Ex, HE, and MA using a predefined DCNN architecture named o-O solution and reported the values of 62.4%, 52.2%, 44.9%, and 31.6% over CWS, Ex, HE, and MA for SE, respectively, which shows a slightly better performance for CWS and Ex than Wang et al. [140] and considerably better on MA than Wang et al. [141]. On the other hand, Wang et al. [141] performed better in HE detection. Further details of these article and others can be found in the Table 5 and Table 6.

## 4. Conclusions

Recent studies for DR detection are mainly focused on automated methods known as CAD systems. In the scope of the CAD system for DR, there are two major approaches known as first classification and staging DR severity and second segmentation of lesions such as MA, HE, Ex, CWS associated with DR.

The DR databases are categorized into public databases (36 out of 43) and private databases (7 out of 43). These databases contain fundus and OCT retinal images, and among these two imaging modalities, fundus photos are used in 86.0% of the published studies. Several public large fundus datasets are available online. The images might have been taken with different systems that affect image quality. Furthermore, some of the image-wise DR labels can be erroneous. The image databases that provide lesion annotations constitute only a small portion of the databases that require considerable resources for pixel-wise annotation. Hence, some of them contain fewer images than image-wise labeled databases. Furthermore, Lesion annotations requires intra-annotator agreement and high annotation precision. These factors make the dataset error sensitive, and its quality evaluation might become complicated.

The DR classification needs a standard grading system validated by clinicians. ETDRS is the gold standard grading system proposed for DR progression grading. Since this grading type needs fine detail evaluation and access to all 7 FOV fundus images, these issues make the use of ETDRS limited. Thus, ICDR with less precise scales is applicable for 1 FOV images to detect the DR severity levels.

The classification and grading DR can be divided into two main approaches, namely, ML-based and DL-based classification. The ML/DL-based DR detection has a generally better performance than ML/DL-based DR grading using the ICDR scale which needs to extract higher-level features associated with each level of DR [57,71]. The evaluation results proved that the DCNN architectures can achieve higher performance scores when large databases are used [72]. There is a trade-off between the performance on one side and the architecture complexity, processing time, and the lack of interpretability over the network’s decisions and extracted features on the other side. Thus, some recent works have proposed semi-DCNN models containing both DL-based and ML-based models acting as classifier or feature extractor [71,72]. The use of regularization techniques is another solution to reduce the complexity of DCNN models [81].

The second approach for CAD-related studies in DR is pixel-wise lesion segmentation or image-wise lesion detection. The main lesions of DR are MA, Ex, HE, CWS. These lesions have a different detection difficulty which directly affects the performance of the proposed pipeline. Among these lesions, the annotation of MA is more challenging [28,167]. Since this lesion is difficult to detect and is the main sign of DR in early stages, some studies focused on the pixel-wise segmentation of this lesion with DCNNs and achieved high enough scores [166]. Although some of the recent DCNN-based works exhibit high performance in term of the standard metrics, the lack of interpretability may not be sufficiently valid for real-life clinical applications. This interpretability brings into the picture the concept of XAI. Explainability studies aim to show the features that influence the decision of a model the most. Singh et al. [87] have reviewed the currently used explainability methods. There is also the need for a large fundus database with high precision annotation of all associated DR lesions to help in designing more robust pipelines with high performance.

## Figures and Tables

**Table 1:** Datasets for DR detection, grading and segmentation and their respective characteristics.

Dataset	No. of Image	Device Used	Access	Country	Year	No. of Subjects	Type	Format	Remarks
DRIVE [29]	40	Canon CR5 non-mydriatic 3CCD camera with a 45° FOV	OA	Netherlands	2004	400	Fundus	JPEG	Retinal vessel segmentation and ophthalmic diseases
DIARETDB0 [30]	130	50° FOV DFC	OA	Finland	2006	NR	Fundus	PNG	DR detection and grading
DIARETDB1 [31]	89	50° FOV DFC	OA	Finland	2007	NR	Fundus	PNG	DR detection and grading
National Taiwan University Hospital [32]	30	Heidelberg retina tomography with Rostock corneal module	OA	Japan	2007–2017	30	Fundus	TIFF	DR, pseudo exfoliation
HEI-MED [33]	169	Visucam PRO fundus camera (Zeiss, Germany)	OA	USA	2010	910	Fundus	JPEG	DR detection and grading
19 CF [34]	60	NR	OA	Iran	2012	60	Fundus	JPEG	DR detection
FFA Photographs & CF [35]	120	NR	OA	Iran	2012	60	FFA	JPEG	DR grading and lesion detection
Fundus Images with Exudates [36]	35	NR	OA	Iran	2012	NR	Fundus	JPEG	Lesion detection
DRiDB [37]	50	Zeiss VISUCAM 200 DFC at a 45° FOV	AUR	Croatia	2013	NR	Fundus	BMP files	DR grading
eOphtha [38]	463	NR	OA	France	2013	NR	Fundus	JPEG	Lesion detection
Longitudinal DR screening data [27]	1120	Topcon TRC-NW65 with a 45 degrees field of view	OA	Netherlands	2013	70	Fundus	JPEG	DR grading
22 HRF [39]	45	CF-60UVi camera (Canon)	OA	Germany and Czech Republic	2013	45	Fundus	JPEG	DR detection
RITE [40]	40	Canon CR5 non-mydriatic 3CCD camera with a 45° FOV	AUR	Netherlands	2013	Same As Drive	Fundus	TIFF	Retinal vessel segmentation and ophthalmic diseases
DR1 [41]	1077	TRC-50× mydriatic camera Topcon	OA	Brazil	2014	NR	Fundus	TIFF	DR detection
DR2 [41]	520	TRC-NW8 retinography (Topcon) with a D90 camera (Nikon, Japan)	OA	Brazil	2014	NR	Fundus	TIFF	DR detection
DRIMDB [42]	216	CF-60UVi fundus camera (Canon)	OA	Turkey	2014	NR	Fundus	JPEG	DR detection and grading
FFA Photographs [43]	70	NR	OA	Iran	2014	70	FFA	JPEG	DR grading and Lesion detection
MESSIDOR 1 [44]	1200	Topcon TRC NW6 non-mydriatic retinography, 45° FOV	OA	France	2014	NR	Fundus	TIFF	DR and DME grading
Lotus eyecare hospital [45]	122	Canon non-mydriatic Zeiss fundus camera 90° FOV	NOA	India	2014	NR	Fundus	JPEG	DR detection
Srinivasan [46]	3231	SD-OCT (Heidelberg Engineering, Germany)	OA	USA	2014	45	OCT	TIFF	DR detection and grading, DME, AMD
EyePACS [47]	88,702	Centervue DRS (Centervue, Italy), Optovue iCam (Optovue, USA), Canon CR1/DGi/CR2 (Canon), and Topcon NW (Topcon)	OA	USA	2015	NR	Fundus	JPEG	DR grading
Rabbani [48]	24 images & 24 videos	Heidelberg SPECTRALIS OCT HRA system	OA	USA	2015	24	OCT	TIFF	Diabetic Eye diseases
DR HAGIS [49]	39	TRC-NW6s (Topcon), TRC-NW8 (Topcon), or CR-DGi fundus camera (Canon)	OA	UK	2016	38	Fundus	JPEG	DR, HT, AMD and Glaucoma
JICHI DR [50]	9939	AFC-230 fundus camera (Nidek)	OA	Japan	2017	2740	Fundus	JPEG	DR grading
Rotterdam Ophthalmic Data Repository DR [51]	1120	TRC-NW65 non-mydriatic DFC (Topcon)	OA	Netherlands	2017	70	Fundus	PNG	DR detection
Singapore National DR Screening Program [52]	494,661	NR	NOA	Singapore	2017	14,880	Fundus	JPEG	DR, Glaucoma and AMD
IDRID [53]	516	NR	OA	India	2018	NR	Fundus	JPEG	DR grading and lesion segmentation
OCTID [54]	500+	Cirrus HD-OCT machine (Carl Zeiss Mediatec)	OA	Multi ethnic	2018	NR	OCT	JPEG	DR, HT, AMD
UoA-DR [55]	200	Zeiss VISUCAM 500 Fundus Camera FOV 45°	AUR	India	2018	NR	Fundus	JPEG	DR grading
APTOS [56]	5590	DFC	OA	India	2019	NR	Fundus	PNG	DR grading
CSME [57]	1445	NIDEK non-mydriatic AFC-330 auto-fundus camera	NOA	Pakistan	2019	NR	Fundus	JPEG	DR grading
OCTAGON [58]	213	DRI OCT Triton (Topcon)	AUR	Spain	2019	213	OCTA	JPEG & TIFF	DR detection
ODIR-2019 [59]	8000	Fundus camera (Canon), Fundus camera (ZEISS), and Fundus camera (Kowa)	OA	China	2019	5000	Fundus	JPEG	DR, HT, AMD and Glaucoma
OIA-DDR [60]	13,673	NR	OA	China	2019	9598	NR	JPEG	DR grading and lesion segmentation
Zhongshan Hospital and First People’s Hospital [61]	19,233	Multiple colour fundus camera	NOA	China	2019	5278	Fundus	JPEG	DR grading and lesion segmentation
AGAR300 [62]	300	45° FOV	OA	India	2020	150	Fundus	JPEG	DR grading and MA detection
Bahawal Victoria Hospital [57]	2500	Vision Star, 24.1 Megapixel Nikon D5200 camera	NOA	Pakistan	2020	500	Fundus	JPEG	DR grading
Retinal Lesions [63]	1593	Selected from EPACS dataset	AUR	China	2020	NR	Fundus	JPEG	DR grading and lesion segmentation
Dataset of fundus images for the study of DR [64]	757	Visucam 500 camera of the Zeiss brand	OA	Paraguay	2021	NR	Fundus	JPEG	DR grading
FGADR [60]	2842	NR	OA	UAE	2021	NR	Fundus	JPEG	DR and DME grading
Optos Dataset (Tsukazaki Hospital) [65]	13,047	200 Tx ultra-wide-field device (Optos, UK)	NOA	Japan	NR	5389	Fundus	JPEG	DR, Glaucoma, AMD, and other eye diseases
MESSIDOR 2 [66]	1748	Topcon TRC NW6 non-mydriatic retinography 45° FOV	AUR	France	NR	874	Fundus	TIFF	DR and DME grading
Noor hospital [67]	4142	Heidelberg SPECTRALIS SD-OCT imaging system	NOA	Iran	NR	148	OCT	TIFF	DR detection

DFC: Digital fundus camera, RFC: Retinal Fundus Camera, FFA: Fundus Fluorescein Angiogram, DR: Diabetic retinopathy, MA: Microaneurysms, DME: Diabetic Macular Edema, FOV: field-of-view, AMD: Age-related Macular Degeneration; OA: Open access AUR: Access upon request; NOA: Not Open access; CF: Colour Fundus; HT: Hypertension; NR: Not Represented.

**Table 2:** Classification-based studies in DR detection using fundus imaging.

Author, Year	Dataset	Grading Details	Pre-Processing	Method	Accuracy	Sensitivity	Specificity	AUC
Abràmoff, 2016 [66]	MESSIDOR 2	Detect RDR and vtDR	No	DCNN: IDx-DR X2.1. ML: RF	NA	96.80%	87.00%	0.98
Chandrakumar, 2016 [90]	EyePACS, DRIVE, STARE	Grade DR based on ICDR scale	Yes	DCNN	STARE and DRIVE: 94%	NA	NA	NA
Colas, 2016 [91]	EyePACS	Grade DR based on ICDR scale	No	DCNN	NA	96.20%	66.60%	0.94
Gulshan, 2016 [69]	EyePACS, MESSIDOR 2	Detect DR based on ICDR scale, RDR and referable DME	Yes	DCNN	NA	EyePACS: 97.5%	EyePACS: 93.4%	EyePACS: 0.99
Wong, 2016 [92]	EYEPACS, MESSIDOR 2	Detect RDR, Referable DME (RDME)	No	DCNN	NA	90%	98%	0.99
Gargeya, 2017 [68]	EyePACS, MESSIDOR 2, eOphtha	Detect DR or non-DR	Yes	DCNN	NA	EyePACS: 94%	EyePACS: 98%	EyePACS: 0.97
Somasundaram, 2017 [76]	DIARETDB1	Detect PDR, NPDR	No	ML: t-SNE and ML-BEC	NA	NA	NA	NA
Takahashi, 2017 [50]	Jichi Medical University	Grade DR with the Davis grading scale (NPDR, severe DR, PDR)	No	DCNN: Modified GoogLeNet	81%	NA	NA	NA
Ting, 2017 [52]	SiDRP	Detect RDR, vtDR, glaucoma, AMD	No	DCNN	NA	RDR: 90.5% vtDR: 100%	RDR: 91.6% vtDR: 91.1%	RDR: 0.93 vtDR: 0.95
Quellec, 2017 [81]	EyePACS, eOphta, DIARETDB 1	Grade DR based on ICDR	Yes	DCNN: L2-regularized o-O DCNN	NA	94.60%	77%	0.955
Wang, 2017 [93]	EyePACS, MESSIDOR 1	Grade DR based on ICDR scale	Yes	Weakly supervised network to classify image and extract high resolution image patches containing a lesion	MESSIDOR 1: RDR: 91.1%	NA	NA	MESSIDOR 1: RDR: 0.957
Benson, 2018 [94]	Vision Quest Biomedical database	Grade DR based on ICDR scale + scars detection	Yes	DCNN: Inception v3	NA	90%	90%	0.95.
Chakrabarty, 2018 [95]	High-Resolution Fundus (HRF) Images	Detect DR	Yes	DCNN	91.67%	100%	100%	F1 score: 1
Costa, 2018 [96]	MESSIDOR 1	Grade DR based on ICDR scale	No	Multiple Instance Learning (MIL)	NA	NA	NA	0.9
Dai, 2018 [97]	DIARETDB1	MA, HE, CWS, Ex detection	Yes	DCNN: Multi-sieving CNN(image to text mapping)	96.10%	87.80%	99.70%	F1 score: 0.93
Dutta, 2018 [98]	EyePACS	Mild NPDR, Moderate NPDR, Severe NPDR, PDR	Yes	DCNN: VGGNet	86.30%	NA	NA	NA
Kwasigroch, 2018 [99]	EyePACS	Grade DR based on ICDR scale	Yes	DCNN: VGG D	81.70%	89.50%	50.50%	NA
Levenkova, 2018 [78]	UWF (Ultra-Wide Field)	Detect CWS, MA, HE, Ex	No	DCNN, SVM	NA	NA	NA	0.80
Mansour, 2018 [72]	EyePACS	Grade DR based on ICDR scale	Yes	DCNN, ML: AlexNet, LDA, PCA, SVM, SIFT	97.93%	100%	0.93	NA
Rajalakshmi, 2018 [7]	Smartphone-based imaging device	Detect DR and vtDR Grade DR based on ICDR scale	No	DCNN	NA	DR: 95.8% vtDR: 99.1%	DR: 80.2% vtDR: 80.4%	NA
Robiul Islam, 2018 [100]	APTOS 2019	Grade DR based on ICDR scale	Yes	DCNN: VGG16	91.32%	NA	NA	NA
Zhang, 2018 [101]	EyePACS	Grade DR based on ICDR scale	Yes	DCNN: Resnet-50	NA	61%	84%	0.83
Zhang, 2018 [102]	EyePACS	Grade DR based on ICDR scale	No	DCNN	82.10%	76.10%	0.855	Kappa score: 0.66
Arcadu, 2019 [103]	7 FOV images of RIDE and RISE datasets	2 step grading based on ETDRS	No	DCNN: Inception v3	NA	66%	77%	0.68
Bellemo, 2019 [104]	Kitwe Central Hospital, Zambia	Grade DR based on ICDR scale	No	DCNN: Ensemble of Adapted VGGNet & Resenet	NA	RDR: 92.25% vtDR: 99.42%	RDR: 89.04%	RDR: 0.973 vt DR: 0.934
Chowdhury, 2019 [105]	EyePACS	Grade DR based on ICDR scale	Yes	DCNN: Inception v3	2 Class: 61.3%	NA	NA	NA
Govindaraj, 2019 [106]	MESSIDOR 1	Detect DR	Yes	Probabilistic Neural Network	98%	Almost 90% from chart	Almost 97% from chart	F1 score: almost 0.97
Gulshan, 2019 [107]	Aravind Eye Hospital and Sankara Nethralaya, India	Grade DR based on ICDR scale	No	DCNN	NA	Aravind: 88.9% SN: 92.1%	Aravind: 92.2% SN: 95.2%	Quadratic weighted K scores: Aravind: 0.85 SN: 0.91
Hathwar, 2019 [108]	EyePACS, IDRID	Detect DR	Yes	DCNN: Xception-TL	NA	94.30%	95.50%	Kappa score: 0.88
He, 2019 [109]	IDRID	Detect DR grade and DME risk	Yes	DCNN: AlexNet	DR grade: 65%	NA	NA	NA
Hua, 2019 [83]	Kyung Hee University Medical Center	Grade DR based on ICDR scale	No	DCNN: Tri-SDN	90.60%	96.50%	82.10%	0.88
Jiang, 2019 [110]	Beijing Tongren Eye Center	DR or Non-DR	Yes	DCNN: Inception v3, Resnet152 and Inception-Resnet-v2	Integrated model: 88.21%	Integrated model: 85.57%	Integrated model: 90.85%	0.946
Li, 2019 [111]	IDRID, MESSIDOR 1	Grade DR based on ICDR scale	No	DCNN: Attention network based on ResNet50	DR: 92.6%, DME: 91.2%	DR: 92.0%, DME: 70.8%	NA	DR: 0.96 DME: 0.92
Li, 2019 [61]	Shanghai Zhongshan Hospital (SZH) and Shanghai First People’s Hospital (SFPH), China, MESSIDOR 2	Grade DR based on ICDR scale	Yes	DCNN: Inception v3	93.49%	96.93%	93.45%	0.9905
Metan, 2019 [112]	EyePACS	Grade DR based on ICDR scale	Yes	DCNN: ResNet	81%	NA	NA	NA
Nagasawa, 2019 [113]	Saneikai Tsukazaki Hospital and Tokushima University Hospital, Japan	Detect PDR	Yes	DCNN: VGG-16	NA	PDR: 94.7%	PDR: 97.2%	PDR: 0.96
Qummar, 2019 [114]	EyePACS	Grade DR based on ICDR scale	Yes	DCNN: Ensemble of (Resnet50, Inception v3, Xception, Dense121, Dense169)	80.80%	51.50%	86.72%	F1 score: 0.53
Ruamviboonsuk, 2019 [115]	Thailand national DR screening program dataset	Grade DR based on ICDR and detect RDME	No	DCNN	NA	DR: 96.8%	DR: 95.6%	DR: 0.98
Sahlsten, 2019 [70]	Private dataset	Detect DR based on multiple grading systems, RDR and DME	Yes	DCNN: Inception-v3	NA	89.60%	97.40%	0.98
Sayres, 2019 [82]	EyePACS	Grade DR based on ICDR	No	DCNN	88.40%	91.50%	94.80%	NA
Sengupta, 2019 [116]	EyePACS, MESSIDOR 1	Grade DR based on ICDR scale	Yes	DCNN: Inception-v3	90. 4%	90%	91.94%	NA
Ting, 2019 [117]	SiDRP, SiMES, SINDI, SCES, BES, AFEDS, CUHK, DMP Melb, with 2 FOV	Grade DR based on ICDR scale	Yes	DCNN	NA	NA	NA	Detect DR: 0.86 RDR: 0.96
Zeng, 2019 [118]	EyePACS	Grade DR based on ICDR scale	Yes	DCNN: Inception v3	NA	82.2%	70.7%	0.95
Ali, 2020 [57]	Bahawal Victoria Hospital, Pakistan.	Grade DR based on ICDR scale	Yes	ML: SMO, Lg, MLP, LMT, Lg employed on selected post-optimized hybrid feature datasets	MLP: 73.73% LMT: 73.00 SLg: 73.07 SMO: 68.60 Lg: 72.07%	NA	NA	MLP: 0.916 LMT: 0.919 SLg: 0.921 SMO: 0.878 Lg: 0.923
Araujo, 2020 [119]	EyePACS, MESSIDOR 2, IDRID, DMR, SCREEN-DR, DR1, DRIMDB, HRF	Grade DR based on ICDR scale	Yes	DCNN	NA	NA	NA	Kappa score: EyePAC: 0.74
Chetoui, 2020 [26]	EyePACS, MESSIDOR 1, 2, eOphta, UoA-DR from the University of Auckland research, IDRID, STARE, DIARETDB0, 1	Grade DR based on ICDR scale	Yes	DCNN: Inception-ResNet v2	97.90%	95.80%	97.10%	98.60%
Elswah, 2020 [74]	IDRID	Grade DR based on ICDR scale	Yes	DCNN: ResNet 50 + NN or SVM	NN: 88% SVM: 65%	NA	NA	NA
Gadekallu, 2020 [71]	DR Debrecen dataset collection of 20 features of MESSIDOR 1	DR or Non-DR	Yes	DCNN ML: PCA + Firefly	97%	92%	95%	NA
Gadekallu, 2020 [120]	DR Debrecen dataset	Detect DR	Yes	ML: PCA+ grey wolf optimization (GWO) + DNN	97.30%	91%	97%	NA
Gayathri, 2020 [121]	MESSIDOR 1, EyePACS, DIARETDB0	Grade DR based on ICDR scale	NA	Wavelet Transform, SVM, RF	MESSIDOR 1: 99.75%	MESSIDOR 1: 99.8%	MESSIDOR 1: 99.9%	NA
Jiang, 2020 [122]	MESSIDOR 1	Image-wise label the presence of MA, HE, Ex, CWS	Yes	DCNN: ResNet 50 based	MA: 89.4% HE: 98.9% Ex: 92.8% CWS: 88.6% Normal: 94.2%	MA: 85.5% HE: 100% Ex: 93.3% CWS: 94.6% Normal: 93.9%	MA: 90.7% HE: 98.6% Ex: 92.7% CWS: 86.8% Normal: 94.4%	MA: 0.94 HE: 1 Ex: 0.97 CWS: 0.97 Normal: 0.98
Lands, 2020 [123]	APTOS 2019, APTOS 2015	Grade DR based on ICDR scale	Ye	DCNN: DensNet 169	93%	NA	NA	Kappa score: 0.8
Ludwig, 2020 [10]	EyePACS, APTOS, MESSIDOR 2, EYEGO	Detect RDR	Yes	DCNN: DenseNet201	NA	MESSIDOR 2: 87%	MESSIDOR 2: 80%	MESSIDOR 2: 0.92
Majumder, 2020 [15]	EyePACS, APTOS 2019	Grade DR based on ICDR scale	Yes	CNN	88.50%	NA	NA	NA
Memari, 2020 [124]	MESSIDOR 1, HEI-MED	Detect DR	Yes	DCNN	NA	NA	NA	NA
Narayanan, 2020 [125]	APTOS 2019	Detect and grade DR based on ICDR scale	Yes	DCNN: AlexNet, ResNe, VGG16, Inception v3	98.4%	NA	NA	0.985
Pao, 2020 [84]	EyePACS	Grade DR based on ICDR scale	Yes	CNN: bichannel customized CNN	87.83%	77.81%	93.88%	0.93
Paradisa, 2020 [73]	DIARETDB 1	Grade DR based on ICDR scale	Yes	ResNet-50 for extraction and SVM, RF, KNN, and XGBoost as classifiers	SVM: 99%, KNN: 100%	SVM: 99%, KNN: 100%	NA	NA
Patel, 2020 [126]	EyePACS	Grade DR based on ICDR scale	Yes	DCNN: MobileNet v2	91.29%	NA	NA	NA
Riaz, 2020 [80]	EyePACS, MESSIDOR 2	NA	Yes	DCNN	NA	EyePACS: 94.0%	EyePACS: 97.0%	EyePAC: 0.98
Samanta, 2020 [127]	EyePACS	Grade DR based on ICDR scale	Yes	DCNN: DenseNet121 based	84.1%	NA	NA	NA
Serener, 2020 [128]	EyePACS, MESSIDOR 1, eOphta, HRF, IDRID	Grade DR based on ICDR scale	Yes	DCNN: ResNet 18	Country: EyePACS: 65% Continent: EyePACS + HRF: 80%	Country: EyePACS: 17% Continent: EyePACS + HRF: 80%	Country: EyePACS: 89% Continent: EyePACS + HRF: 80%	NA
Shaban, 2020 [129]	APTOS	Grade DR to non-DR, moderate DR, and severe DR	Yes	DCNN	88%	87%	94%	0.95
Shankar, 2020 [85]	MESSIDOR 1	Grade DR based on ICDR scale	Yes	DCNN: Histogram-based segmentation + SDL	99.28%	98.54%	99.38%	NA
Singh, 2020 [130]	IDRID, MESSIDOR 1	Grade DME in 3 levels	Yes	DCNN: Hierarchical Ensemble of CNNs (HE-CNN)	96.12%	96.32%	95.84%	F1 score: 0.96
Thota, 2020 [131]	EyePACS	NA	Yes	DCNN: VGG16	74%	80.0%	65.0%	0.80
Wang, 2020 [132]	2 Eye hospitals, DIARETDB1, EyePACS, IDRID	MA, HE, EX	Yes	DCNN	MA: 99.7% HE: 98.4% EX: 98.1% Grading: 91.79%	Grading: 80.58%	Grading: 95.77%	Grading: 0.93
Wang, 2020 [133]	Shenzhen, Guangdong, China	Grade DR severity based on ICDR scale and detect MA, IHE, SRH, HE, CWS, VAN, IRMA, NVE, NVD, PFP, VPH, TRD	No	DCNN: Multi-task network using channel-based attention blocks	NA	NA	NA	Kappa score: Grading: 0.80 DR feature: 0.64
Zhang, 2020 [134]	3 Hospitals in China	Classify to retinal tear & retinal detachment, DR and pathological myopia	Yes	DCNN: InceptionResNetv2	93.73%	91.22%	96.19%	F1 score: 0.93
Abdelmaksoud, 2021 [135]	EyePACS, MESSIDOR 1, eOphta, CHASEDB 1, HRF, IDRID, STARE, DIARETDB0, 1		Yes	U-Net + SVM	95.10%	86.10%	86.80%	0.91
Bora, 2021 [115]	EyePACS	Grade DR based on ICDR scale	No	DCNN: Inception v3	NA	NA	NA	Three FOV: 0·79 One FOV: 0·70
Gangwar, 2021 [136]	APTOS 2019, MESSIDOR 1	Grade DR based on ICDR scale	Yes	DCNN: Inception ResNet v2	APTOS:82.18%MESSIDOR 1: 72.33%	NA	NA	NA
He, 2021 [137]	DDR, MESSIDOR 1, EyePACS	Grade DR based on ICDR scale	No	DCNN: MobileNet 1 with attention blocks	MESSIDOR 1: 92.1%	MESSIDOR 1: 89.2%	MESSIDOR 1: 91%	F1 score: MESSIDOR 1: 0.89
Hsieh, 2021 [32]	National Taiwan University Hospital (NTUH), Taiwan, EyePACS	Detect any DR, RDR and PDR	Yes	DCNN: Inception v4 for any DR and RDR and ResNet for PDR	Detect DR: 90.7% RDR: 90.0% PDR: 99.1%	Detect DR: 92.2% RDR: 99.2% PDR: 90.9%	Detect DR: 89.5% RDR: 90.1% PDR: 99.3%	0.955
Khan, 2021 [138]	EyePACS	Grade DR based on ICDR scale	Yes	DCNN: customized highly nonlinear scale-invariant network	85%	55.6%	91.0%	F1 score: 0.59
Oh, 2021 [2]	7 FOV fundus images of Catholic Kwandong University, South Korea	Detect DR	Yes	DCNN: ResNet 34	83.38%	83.38%	83.41%	0.915
Saeed, 2021 [139]	MESSIDOR, EyePACS	Grade DR based on ICDR scale	No	DCNN: ResNet GB	EyePACS: 99.73%	EyePACS: 96.04%	EyePACS: 99.81%	EyePACS: 0.98
Wang, 2021 [140]	EyePACS, images from Peking Union Medical College Hospital, China	Detect RDR with lesion-based segmentation of PHE, VHE, NV, CWS, FIP, IHE, IRMA and MA, then staging based on ICDR scale	No	DCNN: Inception v3	NA	EyePACS: 90.60%	EyePACS: 80.70%	EyePACS: 0.943
Wang. 2021 [141]	MESSIDOR 1	Grade DR based on ICDR scale	Yes	DCNN: Multichannel-based GAN with semi super- vision	RDR: 93.2%, DR Grading: 84.23%	RDR: 92.6%	RDR: 91.5%	RDR: 0.96

Characteristics and evaluation of DR grading methods. In this table, the methods that have no preprocessing or common preprocessing are mentioned. In addition to the abbreviations described earlier, this table contains new abbreviations: Singapore integrated Diabetic Retinopathy Screening Program (SiDRP) between 2014 and 2015 (SiDRP 14–15), Singapore Malay Eye Study (SIMES), Singapore Indian Eye Study (SINDI), Singapore Chinese Eye Study (SCES), Beijing Eye Study (BES), African American Eye Study (AFEDS), Chinese University of Hong Kong (CUHK), and Diabetes Management Project Melbourne (DMP Melb) and Generative Adversarial Network (GAN).

**Table 3:** Classification-based studies in DR detection using a special preprocessing on fundus images for DR grading in ICDR scale.

Author, Year	Dataset	Pre-Processing Technique	Method	Accuracy
Datta, 2016 [142]	DRIVE, STARE, DIARETDB0, DIARETDB1	Yes, Contrast optimization	Image processing	NA
Lin, 2018 [143]	EyePACS	Yes, Convert to entropy images	DCNN	Original image: 81.8% Entropy images: 86.1%
Mukhopadhyay, 2018 [144]	Prasad Eye Institute, India	Yes, Local binary patterns	ML: Decision tree, KNN	KNN: 69.8%
Pour, 2020 [145]	MESSIDOR 1, 2, IDRID	Yes, CLAHE	DCNN: EfficientNet-B5	NA
Ramchandre, 2020 [146]	APTOS 2019	Yes, Image augmentation with AUGMIX	DCNN: EfficientNetb3, SEResNeXt32x4d	EfficientNetb3: 91.4% SEResNeXt32x4d: 85.2%
Shankar, 2020 [85]	MESSIDOR 1	Yes, CLAHE	DCNN: Hyperparameter Tuning Inception-v4 (HPTI-v4)	99.5%
Bhardwaj, 2021 [147]	DRIVE, STARE, MESSIDOR 1, DIARETDB1, IDRID, ROC	Yes, Image contrast enhancement and OD localization	DCNN: InceptionResNet v2	93.3%
Bilal, 2021 [16]	IDRID	Yes, Adaptive histogram equalization and contrast stretching	ML: SVM + KNN + Binary Tree	98.1%
Elloumi, 2021 [148]	DIARETDB1	Yes, Optic disc location, fundus image partitioning	ML: SVM, RF, KNN	98.4%

AHE: Adaptive Histogram Equalization, CLAHE: Contrast Limited Adaptive Histogram Equalization.

**Table 4:** Classification-based studies in DR detection using OCT and OCTA.

Author, Year	Dataset	Grading Details	Preprocessing	Method	Accuracy	Sensitivity	Specificity	AUC
Eladawi, 2018 [149]	OCTA images, University of Louisville, USA	Detect DR	Yes	ML: Vessel segmentation, Local feature extraction, SVM	97.3%	97.9%	96.4%	0.97
Islam, 2019 [150]	Kermani OCT dataset	NA	Yes	DCNN: DenseNet 201	98.6%	0.986	0.995	NA
Le, 2020 [151]	Private OCTA dataset	Grade DR	No	DCNN: VGG16	87.3%	83.8%	90.8%	0.97
Sandhu, 2020 [75]	OCT. OCTA, clinical and demographical data, University of Louisville Clinical Center, USA	Detect mild and moderate DR	Yes	ML: RF	96.0%	100.0%	94.0%	0.96
Liu, 2021 [77]	Private OCTA dataset	Detect DR	Yes	Logistic Regression (LR), LR regularized with the elastic net (LR-EN), SVM and XGBoost	LR-EN: 80.0%	LR-EN: 82.0%	LR-EN: 84.0%	LR-EN: 0.83

**Table 5:** Segmentation-based studies in DR detection using fundus images.

Author, Year	Dataset	Considered Lesions	Preprocessing	Segmentation Method	Sensitivity/Specificity	AUC
Imani, 2016 [157]	DIARETDB1, HEI-MED, eOphta	Ex	Yes	Dynamic decision thresholding, morphological feature extraction, smooth edge removal	89.01%/99.93%	0.961
Shah, 2016 [154]	ROC	MA	Yes	Curvelet transform and rule-based classifier	48.2%/NA	NA
Quellec, 2017 [81]	EyePACS, eOphta, DIARETDB1	CWS, Ex, HE, MA	Yes	DCNN: o-O solution	DIARETDB1: CWS: 62.4%/NA Ex: 55.2%/NA HE: 44.9%/NA MA: 31.6%/NA	EyePACS: 0.955
Huang, 2018 [155]	MESSIDOR 1, DIARETDB0, 1	NV	Yes	ELM	NA/NA	ACC: 89.2%
Kaur, 2018 [156]	STARE, eOphta, MESSIDOR 1, DIARETDB1, private dataset	Ex, CWS	Yes	Dynamic decision thresholding	94.8%/99.80%	ACC: 98.43%
Lam, 2018 [160]	EyePACS, eOphta	Ex, MA, HE, NV	NA	DCNN: AlexNet, VGG16, GoogLeNet, ResNet, and Inception-v3	NA/NA	EyePACS: 0.99 ACC: 98.0%
Benzamin, 2018 [161]	IDRID	Ex	Yes	DCNN	98.29%/41.35%	ACC: 96.6%
Orlando, 2018 [162]	eOphtha, DIARETDB1, MESSIDOR 1	MA, HE	Yes	ML: RF	NA/NA	0.93
Eftekhari, 2019 [163]	ROC, eOphta	MA	Yes	DCNN: Two level CNN, thresholded probability map	NA/NA	ROC: 0.660
Wu, 2019 [164]	HRF	Blood vessels, optic disc and other regions	Yes	DCNN: AlexNet, GoogleNet, Resnet50, VGG19	NA/NA	AlexNet: 0.94 ACC: 95.45%
Yan, 2019 [165]	IDRID	Ex, MA, HE, CWS	Yes	DCNN: Global and local Unet	NA/NA	Ex: 0.889 MA: 0.525 HE: 0.703 CWS: 0.679
Qiao, 2020 [166]	IDRID	MA	Yes	DCNN	98.4%/97.10%	ACC: 97.8%
Wang, 2021 [141]	EyePACS, images from Peking Union Medical College Hospital	Detect RDR with lesion-based segmentation of PHE, VHE, NV, CWS, FIP, IHE, Ex, MA	No	DCNN: Inception v3 and FCN 32s	PHE: 60.7%/90.9% Ex: 49.5%/87.4% VHE: 28.3%/84.6% NV: 36.3%/83.7% CWS: 57.3%/80.1% FIP: 8.7%/78.0% IHE: 79.8%/57.7% MA: 16.4%/49.8%	NA
Wei, 2021 [63]	EyePACS	MA, IHE, VHE, PHE, Ex, CWS, FIP, NV	Yes	DCNN: Transfer learning from Inception v3	NA/NA	NA
Xu, 2021 [167]	IDRID	Ex, MA, HE, CWS	Yes	DCNN: Enhanced Unet named FFUnet	Ex: 87.55%/NA MA: 59.33%/NA HE: 73.42%/NA CWS: 79.33%/NA	IOU: Ex:0.84 MA: 0.56 HE: 0.73 CWS: 0.75

ICDR scale: International Clinical Diabetic Retinopathy scale, RDR: Referable DR, vtDR: vision threatening DR, PDR: Proliferative DR, NPDR: Non-Proliferative DR, MA: Microaneurysm, Ex: hard Exudate, CWS: Cotton Wool Spot, HE: Hemorrhage, FIP: Fibrous Proliferation, VHE: Vitreous Hemorrhage, PHE: Preretinal Hemorrhage, NV: Neovascularization.

**Table 6:** Segmentation-based studies in DR detection using OCT, OCTA images.

Author, Year	Dataset	Considered Lesions	Pre-Processing	Segmentation Method	Sensitivity	Specificity	AUC
Guo, 2018 [159]	UW-OCTA private dataset	Avascular area	Yes	DCNN	Control: 100.0% Diabetes without DR: 99.0% Mild to moderate DR: 99.0% Severe DR: 100.0%	Control: 84.0% Diabetes without DR: 77.0% Mild to moderate DR: 85.0% Severe DR: 68.0%	ACC:Control: 89.0% Diabetes without DR: 79% Mild to moderate DR: 87% Severe DR: 76.0%
ElTanboly, 2018 [168]	OCT and OCTA images of University of Louisville	12 different retinal layers & segmented OCTA plexuses	No	SVM	NA	NA	ACC: 97.0%
ElTanboly, 2018 [168]	SD-OCT images of KentuckyLions Eye Center	12 distinct retinal layers	Yes	Statistical analysis and extraction of features such as tortuosity, reflectivity, and thickness for 12 retinal layers	NA	NA	ACC: 73.2%
Sandhu, 2018 [169]	OCT images of University of Louisville, USA	12 layers; quantifies the reflectivity, curvature, and thickness	Yes	DCNN: 2 Stage deep CNN	92.5%	95.0%	ACC: 93.8%
Holmberg, 2020 [158]	OCT from Helmholtz Zentrum München, Fundus from EyePACS	Segment retinal thickness map, Grade DR based on ICDR scale	No	DCNN: On OCT: Retinal layer segmentation with Unet On fundus: Self supervised learning, ResNet50	NA	NA	IOU: on OCT: 0.94

## Data Availability

This is review of the published articles, and all the articles are publicly available.
